# An Extended Active-site Motif Controls the Reactivity of the Thioredoxin Fold*[Fn FN2]

**DOI:** 10.1074/jbc.M113.513457

**Published:** 2014-01-27

**Authors:** Despoina A. I. Mavridou, Emmanuel Saridakis, Paraskevi Kritsiligkou, Erin C. Mozley, Stuart J. Ferguson, Christina Redfield

**Affiliations:** From the ‡ Department of Biochemistry, University of Oxford, South Parks Road, Oxford OX1 3QU, United Kingdom and; the § Department of Physical Chemistry, N.C.S.R. Demokritos, Aghia Paraskevi, Athens 15310, Greece

**Keywords:** NMR, Oxidase, Reductase, Thioredoxin, X-ray Crystallography, DsbD, Electrostatics, pKa Values, Thiol-Disulfide Exchange, Thioredoxin Fold

## Abstract

Proteins belonging to the thioredoxin (Trx) superfamily are abundant in all organisms. They share the same structural features, arranged in a seemingly simple fold, but they perform a multitude of functions in oxidative protein folding and electron transfer pathways. We use the C-terminal domain of the unique transmembrane reductant conductor DsbD as a model for an in-depth analysis of the factors controlling the reactivity of the Trx fold. We employ NMR spectroscopy, x-ray crystallography, mutagenesis, *in vivo* functional experiments applied to DsbD, and a comparative sequence analysis of Trx-fold proteins to determine the effect of residues in the vicinity of the active site on the ionization of the key nucleophilic cysteine of the -C*XX*C- motif. We show that the function and reactivity of Trx-fold proteins depend critically on the electrostatic features imposed by an extended active-site motif.

## Teaser

**Background:** Proteins with a thioredoxin (Trx) fold share several structural features but carry out different reactions.

**Results:** The contribution of the electrostatic environment of the active site to the reactivity of Trx-fold proteins is described.

**Conclusion:** Function and reactivity of the Trx fold depend on an extended active-site motif.

**Significance:** These results are expected to assist with functional prediction for Trx-fold proteins.

## Introduction

The cytoplasm of cells is a reducing environment (1) where the formation of disulfide bonds is strictly prevented; glutathione is present in millimolar concentrations and plays a major reductive role. Thioredoxin (Trx),^4^ an essential, ubiquitous protein, acts as an additional source of reductant for the cell. It was identified more than 50 years ago as a cytosolic electron donor (2). Since then, it has been attributed to a wide variety of functions, including reductant provision, protein folding, defense against oxidative stress, apoptosis, host-pathogen interactions, and cell growth, in several cellular locations such as the cytosol, nucleus, and mitochondrion.

**FIGURE 1.**
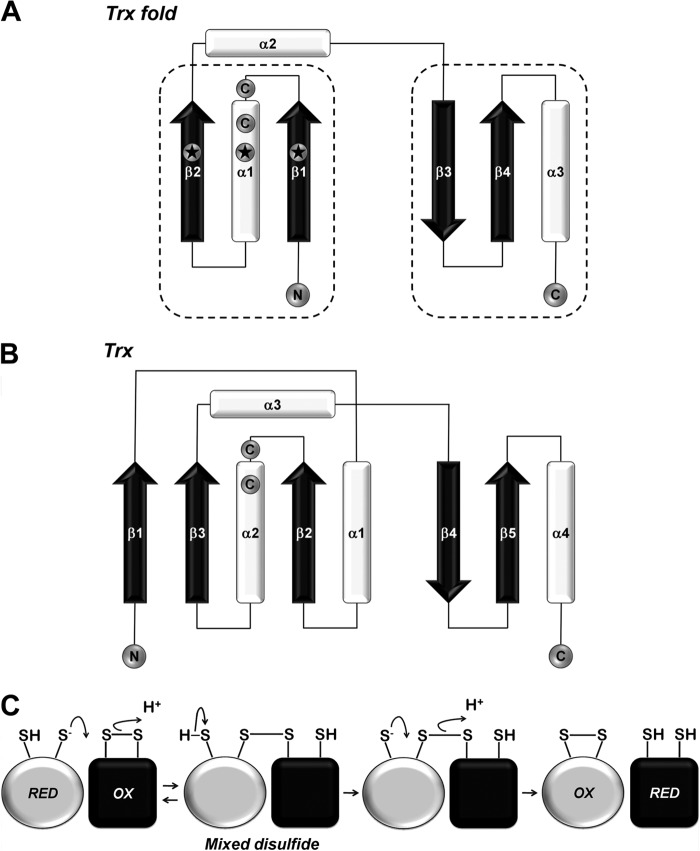
**Schematic representation and comparison of the Trx-fold motif (*A*) with the structure of full-length *E. coli* thioredoxin (*B*).** The Trx fold can be split into two folding units (marked by *dotted lines*) connected by a loop containing an α-helix (helix α2). α-Helices are shown in *white* and β-strands in *black*. The location of the active-site cysteines is indicated (the N-terminal cysteine is located at the end of an α-helix). *Stars* indicate residues within the secondary structure elements that are likely to affect the p*K_a_* value of the N-terminal cysteine. *C,* schematic representation of a thiol:disulfide exchange reaction (bimolecular nucleophilic substitution mechanism). A thiolate anion originating from the deprotonation of a free thiol in the reduced species displaces a sulfur of the disulfide bond in the oxidized species. This results in the formation of a transient mixed-disulfide complex between the two molecules. In a second exchange reaction, the remaining thiolate anion attacks the mixed disulfide bond and resolves it leading to swapping of the initial oxidation states of the two species.

Extracytosolic environments, in contrast with the cell interior, are particularly harsh and oxidizing. A large number of proteins in these environments contain disulfide bonds. These covalent linkages increase protein stability (3, 4) but also, due to their reversible nature, can act as redox switches during signaling (5). The Trx fold (Fig. 1*A*), the main structural element of the Trx superfamily, is abundant in these environments and is involved in oxidative protein folding and electron transfer pathways. This fold is a minimal version of the Trx structure (Fig. 1*B*) (6) that is found in glutaredoxins, protein-disulfide isomerases, oxidases and reductases, glutathione transferases and peroxidases, chloride intracellular channels, and cytochrome *c* maturation proteins (7). The Trx fold is so often present beyond the cytosol because it has proven to be a stable protein scaffold that can carry out oxidation, reduction, or isomerization of disulfide bonds and has the potential to adapt depending on the environment in which it functions.

Proteins with a Trx fold function by participating in thiol-disulfide exchange reactions via their catalytic active-site -C*XX*C- motif (the location of the two cysteines of this motif is indicated in Fig. 1, *A* and *B*). The mechanism of this type of reaction is a bimolecular nucleophilic substitution (Fig. 1*C*). The net result is the oxidation of the original thiol-containing protein (reduced species) and the concomitant reduction of the disulfide-bearing protein (oxidized species). Depending on whether the Trx-fold protein is an oxidase or a reductase/isomerase, it could be either of the two species. The exchange reaction is driven by the reduction potential of the protein initiating the nucleophilic attack (reduced species). However, the rate at which it occurs largely depends on the p*K_a_* value of the nucleophilic cysteine (8) (the N-terminal cysteine of the -C*XX*C- motif) together with other factors like electrostatics of the active site and stereochemical constraints (9, 10). A range of reduction potentials and p*K_a_* values has been reported for Trx-fold proteins in line with their very different roles from strong oxidants (DsbA (11), *E_m_* = −120 mV (12), p*K_a_* = 3.5 (13)) to strong reductants (Trx, *E_m_* = −284 mV (14), p*K_a_* = 7.5 (15)). Numerous studies have focused either on specific characteristics of proteins with a Trx fold that explain their biophysical properties and function or on the comparison of the amino acid sequence of the active-site -C*XX*C- motif that has been proposed to play a significant role in determining the reduction potential and p*K_a_* value of the N-terminal cysteine (16–18). However, it is still not clear how the same protein fold can accommodate such a variety of functions making it difficult to predict the function of new Trx-fold proteins.

The transmembrane thiol:disulfide oxidoreductase DsbD is a three-domain protein responsible for shuttling electrons from the cytoplasm to the oxidizing periplasm of Gram-negative bacteria. Its central domain (tmDsbD) is located in the inner bacterial membrane and is flanked by two periplasmic globular domains (nDsbD, the N-terminal domain, and cDsbD, the C-terminal domain). The transfer of reductive power occurs via sequential thiol-disulfide exchange reactions involving conserved cysteine residues, starting from cytoplasmic Trx and ending with reduced nDsbD (19). nDsbD is the only known oxidoreductase with an immunoglobulin fold (20, 21) and acts as a periplasmic reduction hub (22); it provides electrons to proteins involved in disulfide bond isomerization (Dsb), cytochrome *c* maturation (Ccm) and bacterial conjugation (21, 23–26).

**FIGURE 2.**
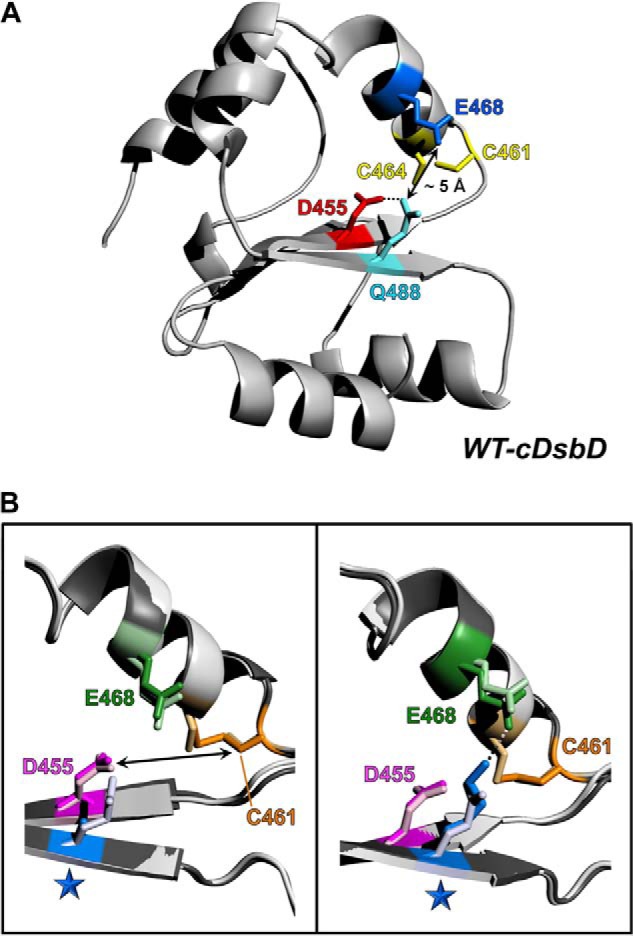
*A,* schematic representation of the three-dimensional structure of wild-type reduced cDsbD (PDB entry 2FWF) (29). Active-site residues Cys-461, Cys-464 (*yellow*), Asp-455 (*red*), Glu-468 (*blue*), and Gln-488 (*cyan*) are shown in *stick* representation. The p*K_a_* value of Cys-461 (N-terminal cysteine of the -C*XX*C- motif) is affected by the proximity of Asp-455 and Glu-468. The carboxylate side chains of these two acidic amino acids are in turn stabilized by the presence of the side chain of Gln-488; the Nϵ2 of the Gln-488 side chain forms a hydrogen bond with the Oδ2 of Asp-455 (shown by a *dotted line*) and is at an ∼5-Å distance from the Oϵ2 of Glu-468. The structure was rendered in PyMOL (82). *B,* superpositions of the active-site regions of wild-type cDsbD_red_ (PDB entry 2FWF) (29) and the structures of the two variants solved in this work Q488A-cDsbD_ox_ (PDB entry 4IP6) on the *left* and Q488K-cDsbD_ox_ (PDB entry 4IP1) on the *right*. The structures were overlaid using the program SuperPose (47) and rendered in PyMOL (82). *Light gray* is used for the chain of wild-type cDsbD_red_ and *dark gray* for the chain of Q488A- or Q488K-cDsbD_ox_; colors used for residues of the cDsbD_ox_ variants were used in a lighter shade for wild-type cDsbD_red_. Active-site residues Cys-461, Cys-464 (*orange*), Asp-455 (*magenta*), and Glu-468 (*green*) are shown in *stick* representation. A *blue star* indicates the position of the Q488A (*left*) and Q488K (*right*) mutations.

cDsbD, like many proteins involved in periplasmic oxidative protein folding, adopts the more conventional Trx fold (27–29). Its role is very specific, to acquire reductant from tmDsbD and transfer it to nDsbD (19). Thus, it acts as a mediator between two non-Trx folds. The reduction of nDsbD by cDsbD has been extensively studied. The x-ray structure of the nDsbD-cDsbD mixed disulfide species has been determined (30), and we have used NMR spectroscopy to examine the active-site properties of cDsbD both in isolation (31) and in complex with nDsbD (32). A schematic representation of the three-dimensional structure of cDsbD is shown in Fig. 2*A*; important residues in the active site are highlighted. We have demonstrated that the p*K_a_* value of the N-terminal cysteine residue (C461) of the cDsbD -C*XX*C- motif is exceptionally high, 10.6, protecting the reduced domain from unwanted side reactions. Upon binding to nDsbD, this p*K_a_* value is lowered in order for reduction of nDsbD to take place. More recently, we have found that the relatively weak affinities of cDsbD for nDsbD are oxidation state-dependent (33). The system is finely tuned to ensure that cDsbD_red_ will form a complex with nDsbD_ox_ so that reductant transfer can occur and that the resulting cDsbD_ox_ and nDsbD_red_ will then dissociate so that they are free to interact with tmDsbD_red_ and DsbC or CcmG, respectively.

The p*K_a_* value of 10.6 found for Cys-461 in cDsbD is the highest reported p*K_a_* for the N-terminal cysteine of the -C*XX*C- motif in thioredoxin family members (18). We have shown by mutagenesis that this high p*K_a_* value results from the close proximity of two conserved acidic residues (Asp-455 and Glu-468) to Cys-461 (31). The chemical shifts of the side chain amide of a glutamine (Gln-488) in the active site also reflect the titration of the carboxyl side chains of Asp-455 and Glu-468 (31). The side chain of Gln-488 forms a hydrogen bond with the side chain of Asp-455 and is in close proximity to the side chain of Glu-468 (Fig. 2*A*) (29). The role of Gln-488 in fine-tuning the reactivity of the active-site cysteines of cDsbD is investigated here using a combination of NMR-based p*K_a_* determination and *in vivo* measurements of the activity of the full-length protein. These are complemented with detailed structural information obtained using x-ray crystallography. The DsbD-specific data along with a comparative sequence analysis of Trx-fold proteins lead us to propose that an extended active-site motif plays an important role in the function of proteins with a Trx fold.

## EXPERIMENTAL PROCEDURES

### 

#### 

##### Construction of Plasmids

Plasmids and oligonucleotides used in this study are listed in supplemental Tables S1 and S2, respectively. The plasmid pDzc1a, described in previous work (31), encodes isolated wild-type cDsbD bearing a thrombin-cleavable C-terminal polyhistidine tag. This construct was used as a template to produce a Q488A and a Q488K variant of isolated cDsbD; site-directed mutagenesis (QuikChange, Qiagen) was performed using oligonucleotides DM1/DM2 and DM3/DM4, respectively. The resulting plasmids were named pDzc10 (Q488A-cDsbD) and pDzc12 (Q488K-cDsbD). pDzc10 was subsequently used as a template to produce a D455N/Q488A variant of cDsbD using oligonucleotides DM5/DM6; the resulting plasmid was named pDzc13.

The plasmid pDsbd1, described in previous work (33), encodes full-length wild-type DsbD bearing a C-terminal streptavidin II tag. This construct was used as a template to produce a Q488A and a Q488K variant of full-length DsbD using oligonucleotides DM1/DM2 and DM3/DM4, respectively. The resulting plasmids were named pDsbd7 (Q488A-DsbD) and pDsbd10 (Q488K-DsbD).

DNA manipulations were conducted using standard methods. KOD Hot Start DNA polymerase (Novagen) was used for all PCRs, and all constructs were sequenced and confirmed to be correct before use. Oligonucleotides were synthesized by Sigma Genosys.

##### Protein Production, Purification, and Characterization

Isolated wild-type cDsbD, Q488A-cDsbD, Q488K-cDsbD, D455N/Q488A-cDsbD, and wild-type nDsbD were expressed using BL21(DE3) cells (supplemental Tables S1 and S3) and were purified from periplasmic extracts of *Escherichia coli* using a C-terminal polyhistidine tag. Production and purification of all proteins were carried out as described in previous work (31, 32) except that 100 μg ml^−1^ ampicillin was used instead of 20 μg ml^−1^ gentamicin. Thrombin cleavage of the affinity tag, when necessary, was performed using the Thrombin CleanCleave kit according to the manufacturer's instructions (Sigma).

Oxidation and reduction of the single disulfide bond in each protein were carried out as follows. 5,5′-Dithiobis-(2-nitrobenzoic acid) (DTNB) was used to oxidize the Cys-103–Cys-109 and Cys-461–Cys-464 disulfide bonds in nDsbD and cDsbD, respectively. 10 mm DTNB was added, and the mixture was incubated at 27 °C for 30 min. Excess DTNB could not be removed completely by simple concentration and re-dilution using a concentration device; proteins were therefore re-purified using their C-terminal polyhistidine or streptavidin II tag, as described previously (33). Disulfide bonds in nDsbD and cDsbD samples were reduced using 10 mm dithiothreitol (DTT). The presence or absence of the Cys-103–Cys-109 and Cys-461–Cys-464 disulfide bonds was confirmed by NMR spectroscopy using the published NMR resonance assignments for nDsbD and cDsbD, respectively (27, 34).

All proteins were subjected to SDS-PAGE and electrospray ionization mass spectrometry (ESI-MS) to confirm that they were pure and of the expected masses. SDS-PAGE analysis was carried out on 10% BisTris NuPAGE gels (Invitrogen) using MES/SDS running buffer prepared according to Invitrogen specifications and including pre-stained protein markers (Invitrogen, SeeBlue Plus 2). ESI-MS was performed using a Micromass Bio-Q II-ZS triple quadrupole mass spectrometer (10-μl protein samples in 1:1 water/acetonitrile, 1% formic acid at a concentration of 20 pmol μl^−1^ were injected into the electrospray source at a flow rate of 10 μl min^−1^). Protein concentrations were determined using the Pierce BCA protein assay kit-reducing agent compatible (Thermo Scientific), following the manufacturer's instructions.

##### AMS Labeling and SDS-PAGE Analysis for Equilibrium Constant Comparison

The effect of the single amino acid substitutions (Q488A and Q488K) on the standard reduction potential of cDsbD was examined by exploiting the direct relationship of this value to the equilibrium constant for the thiol-disulfide exchange reaction of cDsbD with nDsbD. The equilibrium constant for the reaction of nDsbD with wild-type, Q488A-, or Q488K-cDsbD was compared by analysis of the SDS-polyacrylamide gels for the forward thiol-disulfide exchange reaction (starting with nDsbD_ox_ and wild-type, Q488A-, or Q488K-cDsbD_red_) after AMS labeling. Protein concentrations of 0.4 μm were used, and the oxidation state of the protein partners at the start of the reactions was strictly controlled. Each reaction proceeded for 60 min at 25 °C. AMS alkylation of the reaction mixtures was performed by vortexing the protein solutions in 15 mm AMS (Invitrogen), 50 mm Tris-HCl, 3% w/v SDS, and 3 mm EDTA (pH 8.0) for 30 min at 25 °C, followed by a 10-min incubation at 37 °C. SDS-PAGE analysis was carried out using 12% BisTris NuPAGE gels (Invitrogen).

##### In Vivo Studies

The activity of Q488A- and Q488K-DsbD *in vivo* was compared with that of wild-type DsbD. The *E. coli* MC1000 (ΔDsbD) strain (supplemental Table S3) was complemented by plasmids pDsbd1, pDsbd3, pDsbd7, or pDsbd10 (supplemental Table S1) expressing full-length wild-type, C464A-, Q488A-, or Q488K-DsbD, respectively. Cells were also transformed with plasmid pRZ001 (supplemental Table S1), expressing *Paracoccus denitrificans* cytochrome *cd*_1_, the cytochrome *c* chosen to assess the activity of DsbD. The MC1000 parental strain, transformed with pRZ001, served as a positive control. Cell growth and fractionation were performed as described in Ref. 33. Autoinduction was used for expression of all DsbD constructs and of cytochrome *cd*_1_.

SDS-PAGE analysis of the periplasmic fractions was carried out as described above, and gels were stained for covalently bound heme according to the method of Goodhew *et al.* (35). Gel loadings were normalized according to wet cell pellet weights. Densitometry was used to quantify cytochrome *cd*_1_ production using GeneSnap (SYNGENE). The linear relationship between the amount of mature *cd*_1_ present on the gel and the amount detected by densitometry was ensured by using subsaturated loading on the gels (36).

The activity of Q488A- or Q488K-DsbD is expressed as a percentage of the activity of wild-type DsbD, as measured by densitometry. In each experiment, three growths of the variant (Q488A- or Q488K-DsbD) and three growths of wild-type DsbD were carried out, and covalently bound heme was quantified for each growth. Three separate experiments, for bacterial cultures grown on different days, were collected to assess the activity of Q488A-DsbD (Datasets 1–3) and two separate experiments for Q488K-DsbD (Datasets 1 and 2). The individual densitometry readings and the average values for each dataset are reported in Table 1 for the variant and wild-type DsbD. These replicates allow the errors in the reported activity of Q488A- and Q488K-DsbD to be quantified (Table 1).

**TABLE 1:** ***In vivo* activity measurements for wild-type, Q488A- and Q488K-DsbD** Raw data refer to the height of densitometry peaks measured from gels stained for covalently bound heme (Fig. 3). Datasets 1–3 refer to bacterial cultures grown and gels stained on different days. For each dataset, the average of the three densitometry measurements and the standard deviation are reported. Percentage standard errors (100·(σ*_n_*_−1_/√*n*)/mean) are shown in parentheses. Nine replicates were performed for the Q488A-DsbD activity measurement, and six replicates were performed for Q488K-DsbD.

Construct	Dataset 1		Dataset 2		Dataset 3	
	Raw data	Mean and S.D.	Raw data	Mean and S.D.	Raw data	Mean and S.D.
Wild-type DsbD	17,656	20,301 ± 2581 (7%)	19,313	19,792 ± 887 (3%)	19,629	18,781 ± 1271 (4%)
	22,812		20,815		17,319	
	20,434		19,248		19,394	
Q488A-DsbD	15,218	15,714 ± 964 (4%)	14,507	15,565 ± 1238 (5%)	13,210	14,223 ± 925 (4%)
	16,825		15,261		15,024	
	15,098		16,927		14,434	
% Relative activity		77 ± 11		79 ± 7		76 ± 7
Average % relative activity	**77 ± 5**					

Wild-type DsbD	25,817	25,015 ± 2049 (5%)	18,856	18,362 ± 589 (2%)		
	26,542		18,519			
	22,687		17,710			
Q488K-DsbD	25,610	25,579 ± 534 (1%)	22,411	22,422 ± 387 (1%)		
	26,097		22,814			
	25,030		22,041			
% Relative activity		102 ± 9		122 ± 4		
Average % relative activity	**112 ± 5**					

The expression of C464A-, Q488A-, and Q488K-DsbD at comparable levels to that of wild-type DsbD was confirmed by Western blotting of normalized whole cell extracts. Western blotting was carried out as described previously (33).

##### NMR Spectroscopy

The oxidation state of samples for NMR spectroscopy was controlled as described above. Spectra were collected using home-built 500, 600, and 750 MHz NMR spectrometers, controlled with GE/Omega software and equipped with home-built triple-resonance pulsed-field gradient probe heads.

The ^1^H^N^ and ^15^N resonances of oxidized and reduced Q488A- and Q488K-cDsbD were assigned using uniformly ^15^N-labeled samples of 1 mm protein in 95% H_2_O, 5% D_2_O at pH 6.5. Assignments were obtained by comparison of three-dimensional ^15^N-edited NOESY-HSQC spectra for oxidized and reduced Q488A- and Q488K-cDsbD with the spectra of oxidized and reduced wild-type cDsbD (27). The three-dimensional ^15^N-edited NOESY-HSQC spectrum for Q488A-cDsbD was collected at 500 MHz, and data for Q488K-cDsbD was collected at 600 MHz.

NMR experiments for the determination of the p*K_a_* value of the Cys-461 thiol group were performed on identical solutions containing 1 mm [3-^13^C]cysteine-labeled Q488A-, Q488K-, and D455N/Q488A-cDsbD, cleaved of the polyhistidine tag, in 99.9% D_2_O (Sigma). The pH value of the solutions was adjusted by using small volumes of either 0.1 m DCl or 0.1 m NaOD. The pH value of the samples was measured before and after each experiment, and the average of the two measurements was used for data analysis. All pH values given are direct pH-meter readings measured at room temperature and have not been corrected for isotope effects. Two-dimensional ^1^H-^13^C HSQC spectra were collected at 298 K and 750 MHz. Sweep widths of 9345.79 and 4065.04 Hz were used in F_2_ (^1^H) and F_1_ (^13^C), respectively. 44 complex ^13^C increments of 24, 48, or 96 scans were collected with 1024 complex points in the acquisition dimension. NMR data were processed and visualized using NMRPipe, NMRDraw (37), and CCPN analysis (38).

NMR experiments for the determination of the p*K_a_* value of the carboxyl group of Asp-455 were performed using 1 mm samples of ^15^N-labeled Q488A- and Q488K-cDsbD in 95% H_2_O, 5% D_2_O. The pH value of the samples was measured as described above. Two-dimensional ^1^H-^15^N HSQC spectra were collected at 298 K and 500 MHz for Q488A-cDsbD; sweep widths of 6250.00 Hz and 1666.67 Hz were used in F_2_ (^1^H) and F_1_ (^15^N), respectively. Two-dimensional ^1^H-^15^N HSQC spectra were collected at 298 K and 750 MHz for Q488K-cDsbD; sweep widths of 9345.79 Hz and 2500 were used in F_2_ (^1^H) and F_1_ (^15^N), respectively. 128 complex ^15^N increments of 8 or 16 scans were collected with 1024 complex points in the acquisition dimension. Data were processed as described above.

The p*K_a_* values were determined from the ^1^H^β^ and ^13^C^β^ chemical shifts of Cys-461 and Cys-464 and from the ^1^H^N^ and ^15^N chemical shifts of Asp-455 measured as a function of pH. These titration data were fitted to either one- or two-p*K_a_* curves (39) using in-house software. For titrations described by a single p*K_a_* value, the chemical shift at each pH is defined as shown in Equation 1,

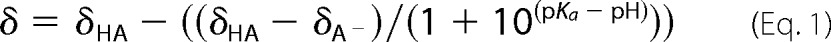
 For titrations described by two p*K_a_* values, the chemical shift at each pH is defined as shown in Equation 2,

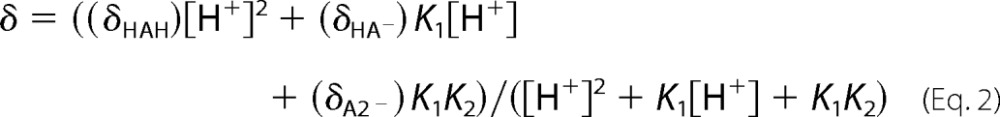
 The p*K_a_* values for Cys-461 and Asp-455 were determined from the fits of at least four resonances in the NMR spectrum (supplemental Tables S4 and S5). The p*K_a_* values for Cys-461 and Asp-455 reported in Table 2 are the average values from more than four resonances, and the errors reported are the standard deviation from the mean. When the pH dependence of a particular NMR resonance is discussed, the p*K_a_* value obtained from the fit of that resonance is reported. When the p*K_a_* of a particular residue is discussed, the average value is reported.

**TABLE 2:** **p*K_a_* values determined for Asp-455 and Cys-461 in wild-type and mutant constructs of cDsbD** The p*K_a_* for Asp-455 was determined in both the reduced (red) and oxidized (ox) protein. The p*K_a_* shown is the average value determined from the fits of several chemical shift curves, and the error is the standard deviation. The number of measurements used for generating the average p*K_a_* values is shown in parentheses. The values reported for reduced Q488A-cDsbD are the microscopic p*K_a_* values.

Construct	Average p*K_a_* value		
	Cys-461 (red)	Asp-455 (red)	Asp-455 (ox)
Wild-type cDsbD	10.6 ± 0.2 (7)	5.8 ± 0.2 (5)	6.7 ± 0.1 (4)
Q488A-cDsbD	7.6 ± 0.2 (5)		8.0 ± 0.3 (7)
	10.4 ± 0.2 (6)		
D455N/Q488A-cDsbD	8.8 ± 0.1 (6)	−	−
Q488K-cDsbD	10.5 ± 0.2 (5)	7.5 ± 0.4 (6)	8.1 ± 0.2 (7)

##### Crystallization, Data Collection, and Structure Determination of Q488A- and Q488K-cDsbD_ox_

Q488A- and Q488K-cDsbD protein solutions were concentrated to 21 mg/ml using 3-kDa cutoff Microcon centrifugal concentration devices (Millipore). Subsequently, cysteine residues were reduced by incubation for 1 h with 3 mm tris(2-carboxyethyl)phosphine (TCEP) as described previously for wild-type cDsbD_red_ (29).

Crystallization trials were set up in vapor diffusion sitting drops, using MRC 96-well crystallization plates (Molecular Dimensions Ltd.) and an OryxNano robotic crystallization system (Douglas Instruments Ltd. (40)) at 4 °C. Drops consisted of 0.75 μl of protein stock to which 0.75 μl of well solution was added. Published conditions (28, 29) did not yield crystals that diffracted well for either mutant even when these conditions were optimized. Several other conditions from ProPlex and one from MemStart crystallization screens (Molecular Dimensions Ltd.) yielded crystals. Crystals of excellent morphology were obtained from the following reservoir conditions: for Q488A-cDsbD, 25% (w/v) PEG 4000, 0.1 m sodium cacodylate, 3 mm TCEP at pH 5.5 (ProPlex HT-96 C9); and for Q488K-cDsbD, 20% (w/v) PEG 4000, 0.1 m sodium citrate, 3 mm TCEP at pH 4.5 (ProPlex HT-96 C2). Crystals appeared after approximately 6 weeks. Although the protein samples were initially in the reduced state and the well solutions contained reducing agent (3 mm TCEP, following the procedure in Ref. 29), the x-ray structures that were determined were of the oxidized proteins.

Crystals were cryoprotected by soaking for a few seconds in solutions containing 20% (v/v) glycerol and 80% (v/v) of their respective well solutions, and they were frozen directly on the nitrogen stream of the x-ray apparatus. Datasets were collected at 100 K with an oscillation angle of 1° (131 frames for Q488A-cDsbD and 122 frames for Q488K-cDsbD) on a Rigaku RU-H3R rotating anode x-ray generator equipped with an R-AXIS IV image plate detector and an Oxford Cryosystems cryostream. Data and refinement statistics are presented in Table 3.

**TABLE 3:** **Crystallographic data and refinement details**

	Q488A-cDsbD	Q488K-cDsbD
**Unit cell**		
Space group	P2_1_2_1_2_1_	P2_1_2_1_2_1_
Cell dimensions	*a* = 34.9, *b* = 51.5, *c* = 84.3 Å	*a* = 35.1, *b* = 51.5, *c* = 85.0 Å

**Data collection**		
Temperature	100 K	100 K
Wavelength	1.5418 Å	1.5418 Å
Resolution (Å)	100 to 2.23 (2.31 to 2.23)*^a^*	100 to 2.47 (2.56 to 2.47)*^a^*
Unique reflections	7224	5345
Redundancy	3.3 (1.6)*^a^*	4.1 (1.9)*^a^*
*R*_sym_	15.5% (38.2%)*^a^*	13.2% (43.3%)*^a^*
Completeness	91.7% (71.2%)*^a^*	89.8% (70.0%)*^a^*
*I*/σ(Ι)	7.5 (2.0)*^a^*	9.1 (2.0)*^a^*
Calculated solvent content	50.5%	52.1%

**Refinement**		
Refinement program	Phenix.refine	Phenix.refine
Resolution	32.6 to 2.23 Å	29.0 to 2.47 Å
Unique reflections	7204	5323
Completeness	91.9%	90.0%
*R* factor	18.3%	19.3%
*R*_free_	25.2%	25.5%
r.m.s.d.*^b^* from ideal bond lengths	0.006 Å	0.003 Å
r.m.s.d. from ideal angles	0.9°	0.7°
Average *B*-factor	19.0 Å^2^	23.3 Å^2^
Non-Gly/Pro residues in most favored regions	91.7% (99/108)	90.7% (98/108)
Non-Gly/Pro residues in additionally allowed regions	8.3% (9/108)	9.3% (10/108)
Non-Gly/Pro residues in disallowed regions	0	0

*^a^* Values in parentheses are for the outermost shell. *R*_sym_ = Σ|*I_h_* − 〈*I_h_*〉|/Σ*I_h_. R*-factor = Σ (|*F*_obs_| − |*F*_calc_|/Σ|*F*_obs_|.

*^b^* r.m.s.d. means root mean square deviation.

For Q488A-cDsbD_ox_, useful data were collected to 2.23 Å resolution. These were processed with the DENZO/SCALEPACK package (41). The crystal belonged to space group P2_1_2_1_2_1_ and contained one molecule per asymmetric unit. Data reduction was performed with the programs Scalepack2mtz and TRUNCATE from the CCP4 program suite (42). Five percent of reflections were flagged for *R*_free_ calculations. A clear molecular replacement solution was found by the program MOLREP (43) using the structure of the wild-type reduced cDsbD (PDB entry 2FWF) (29) as a search model. The program REFMAC (44) was used for the initial stages of structure refinement by the maximum likelihood method. Phenix.refine (45) was used for the later stages, alternating with manual fitting using the program Coot (46). The refinement converged at *R* = 18.3% and *R*_free_ = 25.2%.

For Q488K-cDsbD_ox_, useful data were observed to 2.47 Å resolution. Data processing and refinement steps were largely identical with Q488A-cDsbD_ox_. The crystal also belonged to space group P2_1_2_1_2_1_. The refinement converged at *R* = 19.3% and *R*_free_ = 25.5%.

Both refined models contain residues 428–548 of full-length DsbD. The models contain 136 and 118 water molecules for Q488A-cDsbD and Q488K-cDsbD, respectively. No density was observed before residue 428, after residue 548, or for the side chain atoms beyond the C_β_ of residue 428. No residue lies in the disallowed region of the Ramachandran plot. The coordinates for Q488A-cDsbD and Q488K-cDsbD have been deposited at the Protein Data Bank (PDB accession codes 4IP6 and 4IP1, respectively).

##### Sequence Analysis

Structural overlays were performed using the program SuperPose (47). The following PDB entries were used: *E. coli* thioredoxin (2TRX (48)), *E. coli* cDsbD (2FWF (29)), *E. coli* DsbA (1A2L (49)), *Bacillus subtilis* BdbD (3EU3 (50)), human protein-disulfide isomerase (3UEM (51)), human Trx1 (1TRS (52)), *E. coli* CcmG (2B1K (53)), *B. subtilis* ResA (1SU9 (54)), *B. subtilis* StoA (3ERW (55)), *E. coli* DsbC (1EEJ (56)), and *E. coli* DsbG (1V57 (57)).

## RESULTS

### 

#### 

##### Gln-488 Plays an Important Role in the Function of DsbD in Vivo

**FIGURE 3.**
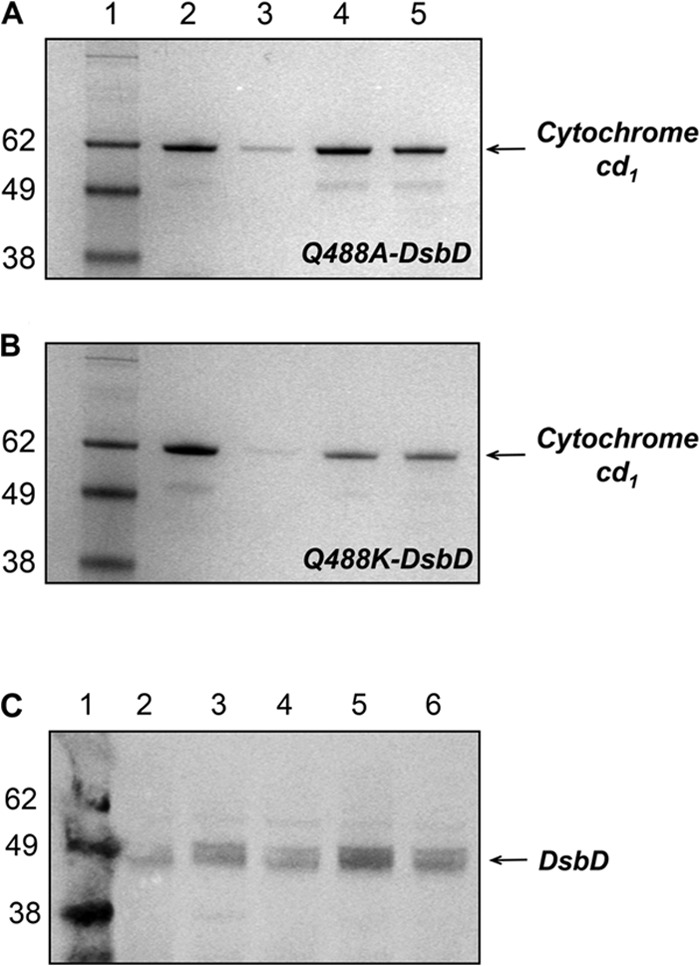
**SDS-PAGE analysis for measuring the activity of full-length Q488A-DsbD (*A*) and Q488K-DsbD (*B*).** Levels of cytochrome *cd*_1_ production under anaerobic growth conditions are assessed by staining and detecting covalently bound heme. *Lane 1* shows molecular mass markers (as indicated, in kDa); *lane 2* shows the level of *cd*_1_ production in the parental strain; *lanes 3–5* show the level of *cd*_1_ production in the *dsbD* deletion strain complemented with full-length C464A-DsbD (*lane 3*), wild-type DsbD (*lane 4*), and Q488A-DsbD (*lane 5* (*A*)) or Q488K (*lane 5* (*B*)) on a plasmid. *C,* SDS-PAGE analysis for the assessment of the expression level of wild-type and variants of full-length DsbD during anaerobic cell culture. Western blotting with an anti-cDsbD antibody was used. *Lane 1* shows molecular mass markers (as indicated in kDa). *Lane 2* shows the level of full-length wild-type DsbD production in the parental strain, and *lanes 3–6* show the levels of C464A-DsbD (*lane 3*), wild-type DsbD (*lane 4*), Q488A-DsbD (*lane 5*), and Q488K-DsbD (*lane 6*) production from a plasmid in the *dsbD* deletion strain.

We employed mutagenesis to determine the importance of Gln-488 for the activity of full-length DsbD. Heme attachment to *c*-type cytochromes in the periplasm requires the cysteine residues of the apocytochrome to be reduced via a pathway involving DsbD (58). We have developed an *in vivo* assay where the production of *c*-type cytochromes is used to assess the activity of full-length DsbD (33). A *dsbD* deletion strain was complemented with plasmids expressing full-length wild type, C464A-, and Q488A-DsbD. The activity of full-length Q488A-DsbD was compared with that of the wild-type protein by measuring the levels of mature cytochrome *cd*_1_ produced in the *E. coli* periplasm. Fig. 3*A* shows the SDS-PAGE analysis of the periplasmic extracts, where cytochrome *cd*_1_ is detected by staining the gel for covalently bound heme. Expression of C464A-DsbD, which is inactive as it lacks one of the six essential cysteines (59), results in very low levels of *cd*_1_ production (Fig. 3*A, lane 3*) (60). When the cells are complemented with Q488A-DsbD (Fig. 3*A, lane 5*) the level of *cd*_1_ maturation is 23% lower than observed for the wild-type protein (*lane 4*) (Table 1).

A Western blot showing the expression levels of full-length DsbDs is shown in Fig. 3*C*. The amount of wild-type DsbD in the parental strain (Fig. 3*C, lane 2*) is lower than that in the *dsbD* deletion strain complemented with wild-type DsbD on a plasmid (*lane 4*). Wild-type DsbD, C464A-DsbD, and Q488A-DsbD express at comparable levels (Fig. 3*C*, *lanes 4, 3,* and *5*, respectively); therefore, the drop in activity of Q488A-DsbD *in vivo* is due to the Q488A substitution and not to overall lower levels of DsbD expression.

##### Gln-488 Contributes to the Biophysical Properties of Isolated cDsbD

**FIGURE 4.**
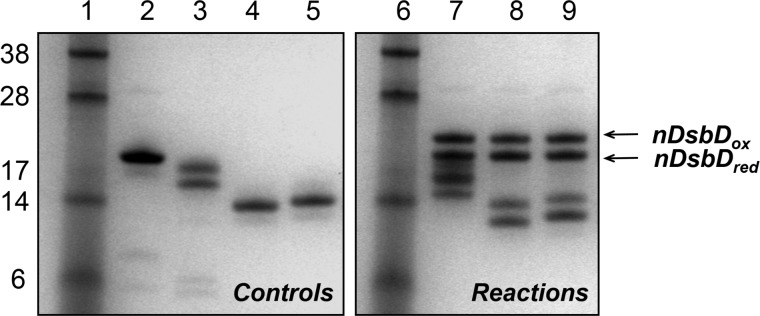
**SDS-PAGE analysis for comparison of the equilibrium constants for the reaction of nDsbD_ox_ with wild-type cDsbD_red,_ Q488A-cDsbD_red_, or Q488K-cDsbD_red_.** *Lanes 1* and *6* show molecular mass markers. *Lanes 2–5* and *7–9* show protein samples after AMS alkylation. *Lanes 2* and *3* show wild-type nDsbD_ox_ and cDsbD_red_ (this construct runs as a diffuse double band on SDS-PAGE because its PelB signal sequence, which targets the protein to the periplasm, is cleaved inefficiently by the signal peptidase, leaving a large fraction of the protein uncleaved (confirmed by mass spectrometry and N-terminal sequencing)), respectively. *Lanes 4* and *5* show Q488A-cDsbD_red_ and Q488K-cDsbD_red_, respectively. *Lane 7* shows the reaction nDsbD_ox_ with wild-type cDsbD_red_; *lane 8* shows the reaction of nDsbD_ox_ with Q488A-cDsbD_red_, and *lane 9* shows the reaction of nDsbD_ox_ with Q488K-cDsbD_red_. The bands corresponding to wild-type, Q488A-cDsbD, and Q488K-cDsbD in *lanes 7–9* have different appearances due to differences in the extent of processing of the PelB signal sequence, which is a variable between cultures and not possible to control.

A plausible explanation for the difference in reactivity of Q488A-DsbD compared with the wild-type protein could be that the Q488A substitution altered the reduction potential of cDsbD. An AMS labeling experiment was performed as described above under “Experimental Procedures.” The equilibrium constants of the reaction of oxidized nDsbD (nDsbD_ox_) with wild-type reduced cDsbD (cDsbD_red_) or Q488A-cDsbD_red_ were compared. The relative intensities of the bands for nDsbD_ox_ and nDsbD_red_, shown in Fig. 4, are the same for reactions with wild-type cDsbD (*lane 7*) and Q488A-cDsbD (*lane 8*) indicating that the equilibrium constants for the two reactions are comparable and thus the reduction potential of cDsbD is not affected by the Q488A substitution.

**FIGURE 5.**
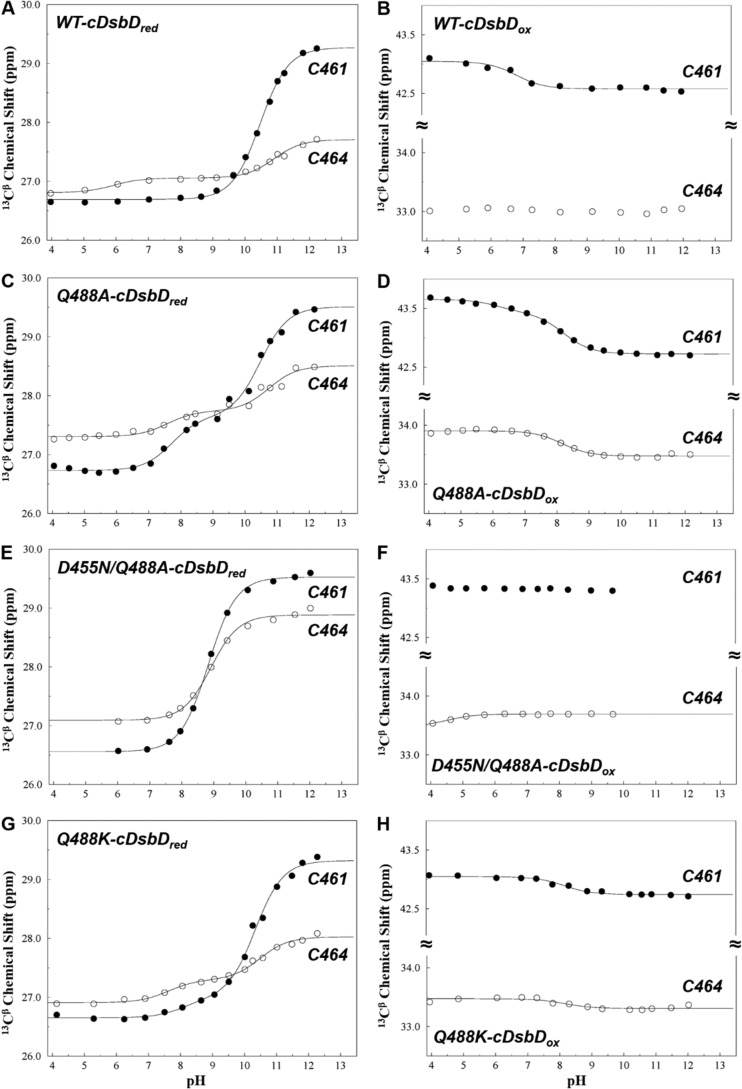
**pH dependence of the ^13^C^β^ peaks from Cys-461 and Cys-464 in ^13^C^β^-cysteine-labeled wild-type, Q488A-, D455N/Q488A-, and Q488K-cDsbD.** *A, C, E,* and *G* are for reduced wild-type cDsbD and variants. *B, D, F,* and *H* are for oxidized wild-type cDsbD and variants. *Closed circles* are used for ^13^C^β^ chemical shifts of Cys-461, and *open circles* are used for ^13^C^β^ chemical shifts of Cys-464. The *continuous lines* show the best fit to a single p*K_a_* value or to two p*K_a_* values for each dataset. The fitting procedure is described under “Experimental Procedures,” and the fitted parameters are summarized in supplemental Table S4. Analysis of the ^1^H^β^ chemical shifts gives similar p*K_a_* values (not shown).

The drop in Q488A-DsbD activity *in vivo* could also be explained by a difference in the rate of reduction of nDsbD_ox_ by Q488A-cDsbD_red_. This would be related to a difference in the p*K_a_* value of the attacking Cys-461 in Q488A-DsbD compared with the wild-type domain. We measured the p*K_a_* value of Cys-461 in Q488A-cDsbD using two-dimensional NMR spectroscopy. Changes in the ^13^C^β^ chemical shifts of the cysteine residues in the ^1^H-^13^C HSQC spectrum of reduced and oxidized Q488A-cDsbD were measured as a function of pH, at pH values ranging from 4 to 13, as shown in Fig. 5, *C* and *D* (*closed circles*), respectively. These titrations reveal a very different behavior to that seen for wild-type cDsbD (Fig. 5, *A* and *B*).

For wild-type cDsbD_red_ the ^13^C^β^-^1^H^β^ peaks of Cys-461 show significant chemical shift changes above pH 9 (Fig. 5*A*) that yield a p*K_a_* value of 10.5. There is no change in the ^13^C^β^ chemical shifts of Cys-461 for the oxidized protein above pH 9 (Fig. 5*B*). The latter, along with the large downfield shift (∼2.5 ppm) with increasing pH that is characteristic of the deprotonation of a cysteine thiol group observed for cDsbD_red_, indicates ionization of Cys-461 with a p*K_a_* value of 10.6 ± 0.2 (31). In Q488A-cDsbD_red_, the ^13^C^β^ chemical shifts of Cys-461 also show a large downfield shift (∼2.7 ppm in total) above pH 7 (Fig. 5*C*). However, fitting of the titration data produced a double sigmoid curve with two almost equal steps that give p*K_a_* values of 7.7 and 10.5 (supplemental Table S4). ^13^C^β^ chemical shifts of Cys-461 in Q488A-cDsbD_ox_ also change above pH 7, with an upfield shift of ∼0.7 ppm, and this change can be fitted to a p*K_a_* value of 8.2 (supplemental Table S4). In wild-type cDsbD_red_, the pH-dependent chemical shift change observed for ^13^C^β^ of Cys-464 (Fig. 5*A*, *open circles*) was assigned previously, using mutagenesis, to the “through-space” electrostatic effect of the deprotonation of the Cys-461 thiol on Cys-464 ^13^C^β^, rather than the titration of Cys-464 itself (31). This was also observed in Q488A-cDsbD (Fig. 5*C*, *open circles*); smaller changes in chemical shifts for the ^13^C^β^ of Cys-464 yields two p*K_a_* values (7.6 and 10.7) that are consistent with the values measured from Cys-461 ^13^C^β^. Cys-464 in Q488A-cDsbD_ox_ shows a pH-dependent ^13^C^β^ shift with a p*K_a_* value of 8.1; this is in contrast to wild-type cDsbD_ox_ where Cys-464 does not shift with pH.

The titration profile of Cys-461 in Q488A-cDsbD resembles that of the nucleophilic cysteine (Cys-32) of *E. coli* thioredoxin. Measurement of the p*K_a_* value of this residue has not been straightforward because the complex titration behavior recorded for Cys-32 required the invocation of microscopic p*K_a_* values (7.5 and 9.2) for two interacting active-site residues, Asp-26 and the N-terminal Cys-32 (15, 61). In a specific thioredoxin molecule, if Asp-26 loses its side chain proton with a p*K_a_* value of 7.5, then the p*K_a_* value of Cys-32 in this molecule will be increased to 9.2. In contrast, if Cys-32 loses its thiol proton with a p*K_a_* of 7.5, then Asp-26 in that molecule will have the higher p*K_a_* value of 9.2. In cDsbD, Asp-455 is structurally equivalent to Asp-26 of thioredoxin (Fig. 7), just six amino acids before the N-terminal cysteine of the -C*XX*C- motif, and it has been shown to play a major role in the elevated p*K_a_* value of Cys-461 (31). However, wild-type cDsbD does not show microscopic p*K_a_* behavior; in cDsbD_red_, Asp-455 titrates first with a p*K_a_* value of 5.8 ± 0.2 followed by titration of Cys-461 with a p*K_a_* of 10.6 ± 0.2. The replacement of Gln-488 by an alanine may perturb the active site of cDsbD leading to a stronger coupling of these p*K_a_* values.

##### Q488A Mutation Leads to Microscopic pK_a_ Values between Asp-455 and Cys-461 in Q488A-cDsbD

**FIGURE 6.**
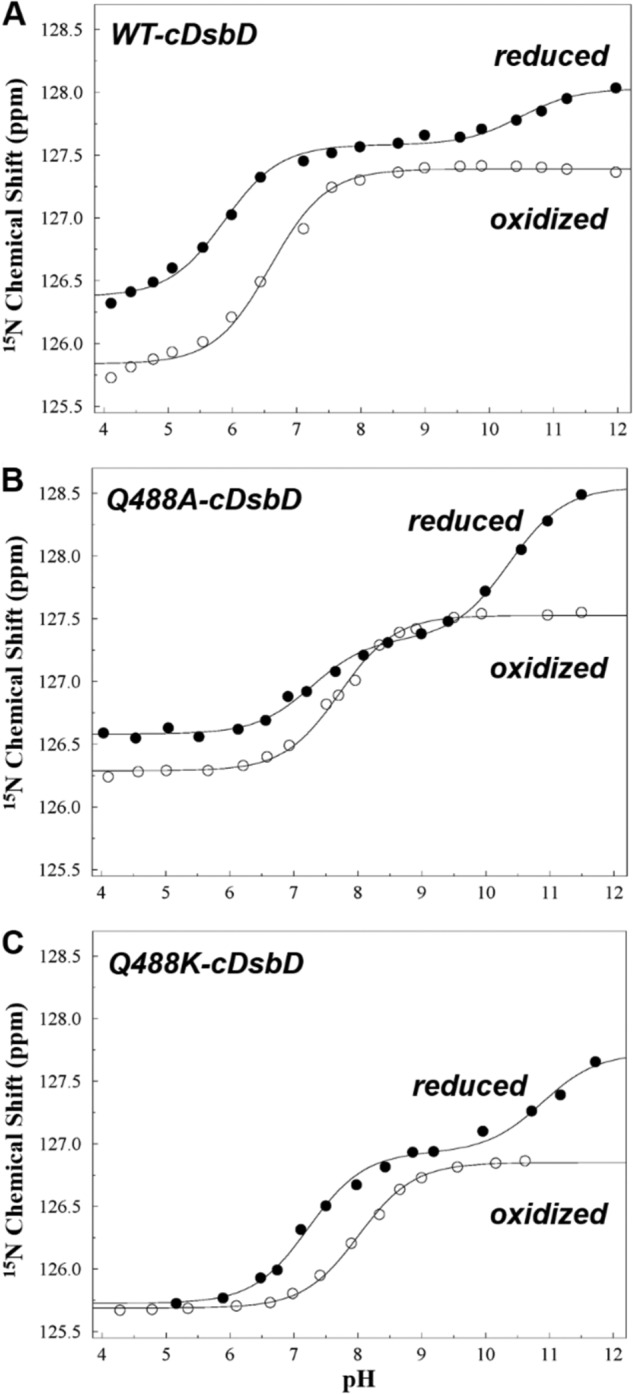
**Measurement of the p*K_a_* value of Asp-455 in the active site of ^15^N-labeled wild-type (*A*), Q488A- (*B*), and Q488K-cDsbD (*C*).** ^15^N chemical shifts of the backbone amide of Asp-455 were plotted as a function of pH for the oxidized (○) and reduced (●) proteins. The *continuous lines* show the best fit to a single p*K_a_* value or to two p*K_a_* values for each dataset. The fitting procedure is described under “Experimental Procedures,” and the fitted parameters are summarized in supplemental Table S5. Analysis of the ^1^H^N^ chemical shifts gives similar p*K_a_* values (not shown).

We have previously determined the p*K_a_* value of the buried Asp-455 residue in wild-type cDsbD (31) as 6.7 ± 0.2 for cDsbD_ox_ and 5.8 ± 0.2 for cDsbD_red_ by monitoring the ^15^N chemical shifts of its backbone amide (Fig. 6*A* and Table 2). A large chemical shift change is observed at low pH corresponding to the deprotonation of Asp-455. A much smaller shift change is observed in cDsbD_red_ at higher pH due to the influence of deprotonation of Cys-461. If the ionization behavior of Asp-455 and Cys-461 in Q488A-cDsbD is characterized by microscopic p*K_a_* values, then the titration curve for Asp-455 in the reduced protein should show the same double sigmoidal pattern observed for Cys-461. For Q488A-cDsbD_red_ (Fig. 6*B*, *closed circles*), the pH dependence of the ^15^N chemical shift of the backbone amide of Asp-455 can be fitted to two p*K_a_* values of 7.3 and 10.4, showing almost equal chemical shift changes for the two transitions.

If either Asp-455 or Cys-461 is replaced by a nontitrating residue, then the microscopic p*K_a_* behavior should disappear and simple titration curves should be observed. Oxidized Q488A-cDsbD contains the Cys-461–Cys-464 disulfide bond and therefore lacks any titratable thiol groups. A single p*K_a_* value of 7.7 was measured for Asp-455 in Q488A-cDsbD_ox_ (Fig. 6*B*, *open circles*, and supplemental Table S5). The side chain carboxyl group of Asp-455 is absent in the D455N/Q488A-cDsbD double variant. Both Cys-461 and Cys-464 show a large downfield shift (∼3.0 ppm for Cys-461 and ∼1.8 ppm for C464) (Fig. 5*E*) in D455N/Q488A-cDsbD_red_, which yields a single p*K_a_* value of 8.8 ± 0.1 (Table 2). Once more, we conclude that the titration of Cys-464 reflects the through-space effect of the ionization of Cys-461. These results for Q488A-cDsbD_ox_ and D455N/Q488A-cDsbD_red_ reinforce our conclusion that Asp-455 and Cys-461 are strongly coupled through microscopic p*K_a_* values in Q488A-cDsbD.

The phenomenon of microscopic p*K_a_* values between Asp-455 and Cys-461 would have important implications for the function of Q488A-DsbD. In a given population of Q488A-cDsbD_red_ protein molecules, some molecules will have a p*K_a_* of 7.6 for Asp-455 and a p*K_a_* of 10.4 for Cys-461, a situation similar to that observed for wild-type cDsbD. Other Q488A-cDsbD_red_ molecules will have a p*K_a_* of 7.6 for Cys-461 and a p*K_a_* of 10.4 for Asp-455. This dramatically lower cysteine p*K_a_* would make Q488A-cDsbD_red_ more susceptible to reoxidation (via the action of DsbA or molecular oxygen) and would decrease its effectiveness at reducing nDsbD. This could explain the reduced amounts of cytochrome *cd*_1_ produced in the presence of the Q488A-DsbD variant compared with wild-type protein, because a percentage of the protein molecules can undergo a futile cycle and not fulfill their normal function of reducing nDsbD.

##### Role of Gln-488 Can Be Performed by Other Amino Acids

The above results clearly indicate an important role for Gln-488 in the electrostatic control of the active site of cDsbD and the reactivity of the full-length protein *in vivo*. However, this residue is not strictly conserved in cDsbD; among 494 sequences examined, only 380 contain a glutamine (∼78% conservation). In the remaining sequences, residue 488 is usually an arginine (72 organisms) and sometimes a lysine (42 organisms). It is interesting to note that the side chain of Lys-57 of *E. coli* thioredoxin overlays closely with the side chain of Gln-488 of cDsbD. The side chains of Asp-26 (structurally equivalent to Asp-455 in cDsbD) and Lys-57 are involved in a buried salt-bridged/hydrogen-bonded interaction, and Lys-57 has been shown to play an important role in the regulation of the ionization state of Asp-26, and therefore indirectly of the active-site thiols, in thioredoxin (62). Stabilization of the fully buried Asp-26 side chain anion by this noncovalent interaction with Lys-57 lowers its p*K_a_* value and leads to microscopic p*K_a_* values between Asp-26 and Cys-32 in thioredoxin.

We chose to study the Q488K variant of isolated cDsbD and full-length DsbD because of the key role of Lys-57 in *E. coli* thioredoxin. The ^13^C^β^ chemical shifts of Cys-461 and Cys-464 and the ^15^N chemical shifts of the backbone amide of Asp-455 were measured as a function of pH for both oxidation states of Q488K-cDsbD (Figs. 5, *G* and *H,* and 6*C*). The observed titration curves are very similar to those observed for wild-type cDsbD (Figs. 5, *A* and *B,* and 6*A*); although the titration curves of Asp-455 and Cys-461 show that these residues influence each other's chemical shifts, they do not show the hallmarks of microscopic p*K_a_* values observed for Q488A-cDsbD. A p*K_a_* value of 10.5 ± 0.2 was observed for Cys-461 in Q488K-cDsbD_red_ (Fig. 5*G*, *closed circles*, supplemental Table S4, and Table 2); this is very close to the wild-type value of 10.6 ± 0.2. Plots of the chemical shifts of the Asp-455 backbone ^15^N gave a p*K_a_* value of 8.1 ± 0.2 for Asp-455 in Q488K-cDsbD_ox_ and a value of 7.5 ± 0.2 in Q488K-cDsbD_red_; the latter titration curve also shows the influence at high pH of the titration of Cys-461. These p*K_a_* values for Asp-455 are higher than the values of 6.7 ± 0.1 and 5.8 ± 0.2 observed in wild-type DsbD. Nevertheless, in Q488K-cDsbD, the side chain carboxyl group of Asp-455 undergoes deprotonation at a lower pH value than the thiol of Cys-461.

Replacement of Gln-488 by a lysine did not alter the p*K_a_* value of the attacking Cys-461, implying that this variant should be equivalent to wild-type DsbD *in vivo*. We tested this as above, comparing the cytochrome *cd*_1_ levels in periplasmic fractions of a *dsbD* deletion strain that was complemented with plasmids expressing full-length wild-type, C464A-, and Q488K-DsbD. Results can be seen in Fig. 3*B* and Table 1. When the cells were complemented with Q488K-DsbD (Fig. 3*B, lane 5*) the level of *cd*_1_ maturation is ∼10% higher than observed for the wild-type protein (*lane 4*). The overall levels of expression of full-length DsbD constructs were checked by Western blotting (Fig. 3*C*, compare *lanes 4* and *6*) and were found to be comparable (with Q488K-DsbD being possibly a bit less abundant than wild-type DsbD). The effect of the Q488K substitution on the reduction potential of cDsbD was also assessed (Fig. 4, compare the intensities of bands for nDsbD in *lanes 7* and *9*) and was found to be negligible. Therefore, we can conclude that Q488K-cDsbD has similar biophysical properties to the wild-type domain, and Q488K-DsbD functions equally effectively as wild-type DsbD *in vivo*.

##### Active-site Properties of cDsbD Variants Can Be Explained Using Atomic Level Information

In this work, the structures of Q488A- and Q488K-cDsbD have been determined by x-ray diffraction at a resolution of 2.23 and 2.47 Å, respectively (PDB entries 4IP6 for Q488A-cDsbD and 4IP1 for Q488K-cDsbD). Despite the presence of reducing agent (TCEP) in the mother liquor, both proteins were found to be oxidized with a Cys-461–Cys-464 disulfide bond clearly present. X-ray structures have been determined previously for both oxidation states of wild-type cDsbD (PDB entries 2FWE and 2FWF, respectively (29)). Oxidized and reduced cDsbD show no significant structural change apart from a reorientation of the cysteine side chains in the active site. The distance between Nϵ2 of Gln-488 and Oδ2 of Asp-455 or Oϵ2 of Glu-468 remains almost identical in the two oxidation states of wild-type cDsbD. This allows us to use the x-ray structures of Q488A- and Q488K-cDsbD_ox_ to rationalize the impact of the Gln-488 substitutions on the active-site properties of cDsbD.

Structural overlays of the active site of wild-type cDsbD_red_ and Q488A-cDsbD_ox_ (*left panel*) or Q488K-cDsbD_ox_ (*right panel*) are shown in Fig. 2*B*. The cysteine pair (Cys-461 and Cys-464), Asp-455, and Glu-468 of wild-type cDsbD overlay well with the same residues of Q488A-cDsbD (Fig. 2*B*, *left panel*). Cys-461 and Cys-464 have the same orientation in both structures despite the fact that the two domains were crystallized in different oxidation states. The only difference is that the side chain of Gln-488 is absent in Q488A-cDsbD and replaced by the much shorter alanine side chain. The carboxyl side chains of Asp-455 and Glu-468 in both wild-type and Q488A-cDsbD are oriented toward Cys-461 and remain in close proximity (<∼8 Å) to the N-terminal cysteine of the -C*XX*C- motif. The side chain solvent accessibilities of Asp-455 (9%) and Glu-468 (57%) in Q488A-cDsbD are comparable with those in wild-type cDsbD (7% for Asp-455 and 42% for Glu-468). In both proteins, the more accessible side chain of Glu-468 has a near normal p*K_a_* value (lower than 5). The almost complete burial of Asp-455 leads to an elevated p*K_a_* value for this residue. In wild-type cDsbD, the hydrogen bond interaction with Gln-488 (Fig. 2*A*) stabilizes the carboxylate group of Asp-455 and results in a p*K_a_* value of 5.8 or 6.7, depending on the oxidation state of the protein. In Q488A-cDsbD, the absence of this stabilizing interaction leads to a further elevation of the p*K_a_* value of Asp-455 to a point where it becomes indistinguishable from the p*K_a_* value of Cys-461. Thus, Cys-461 and Asp-455 share the same p*K_a_* values (7.6 and 10.4).

Cys-461, Cys-464, Asp-455, and Glu-468 of Q488K-cDsbD overlay well with the same residues of wild-type cDsbD (Fig. 2*B*, *right panel*). However, unlike *E. coli* thioredoxin where Lys-57 forms a salt bridge with Asp-26 (equivalent to Asp-455 in cDsbD) (62), Lys-488 is in close proximity to Glu-468, and the two residues have hydrogen bond interactions with a bridging water molecule. The *N*^z^ of Lys-488 has a high solvent accessibility (36%), which makes it possible to interact with the solvent-accessible Glu-468 (side chain solvent accessibility 36%). Asp-455, which in the wild-type domain forms a hydrogen bond to the side chain of Gln-488, is just hydrated in Q488K-cDsbD but still remains almost completely buried (side chain solvent accessibility 6%) leading to an elevated p*K_a_* of 7.5. The *N*^z^ of Lys-488 is more than 11 Å away from the thiol group of Cys-461; therefore, this positive charge does not affect the p*K_a_* value of Cys-461. Although Asp-455 does not interact directly with the charged side chain of Lys-488, the side chains of these two residues are within a distance of 5–6 Å. The closer proximity of Lys-488 to Asp-455 ensures that this residue has a lower p*K_a_* value (7.5) than observed for Cys-461 (10.5). In accordance with this, full-length Q488K-DsbD is fully functional *in vivo* and as effective as the wild-type protein.

## DISCUSSION

### 

#### 

##### Active Site of cDsbD Is Finely Tuned

**FIGURE 7.**
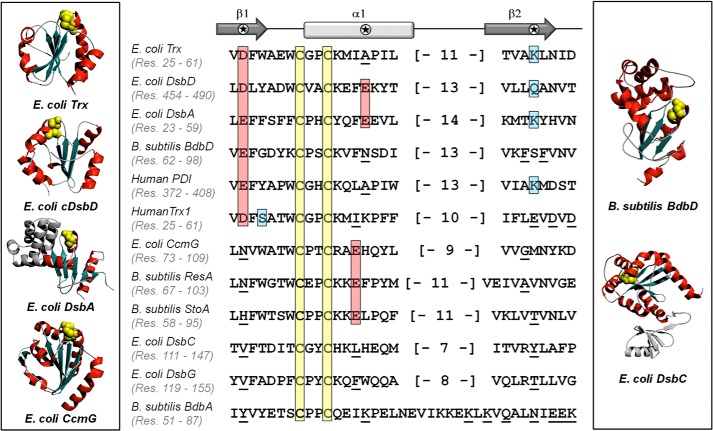
**Comparative sequence analysis of the extended active-site motif of Trx-fold proteins from different organisms.** The alignment was done manually, using the -C*XX*C- motif as well as secondary structural elements as a reference point. Active-site cysteines are highlighted in *yellow,* and acidic residues that can affect the p*K_a_* value of the attacking cysteine are shown in *red. Cyan* is used to indicate amino acids that could form a hydrogen bond or salt bridge with key acidic residues of the active site. Residues that are in structurally equivalent positions to highlighted residues but that are not thought to affect the p*K_a_* value of the attacking cysteine are *underlined*. The *numbers in brackets* refer to the number of amino acids belonging to the end of helix α1 and the loop connecting helix α1 and strand β2, which are not shown. The structure and function of *B. subtilis* BdbA are still unknown. In this case, residues that could possibly compose the extended active site are *underlined*. The secondary structure elements of the Trx fold are displayed above the primary sequences of the selected proteins. Residues affecting the p*K_a_* value of the attacking cysteine can be found in helix α1 and strands β1 and β2 (indicated by a *star*). The panels flanking the sequence alignment show the structural diversity of Trx-fold proteins; *E. coli* thioredoxin, cDsbD, and CcmG are classical Trx-fold proteins; DsbA has a modified Trx fold with an inserted helical domain; *B. subtilis* BdbD has a calcium-binding Trx fold, and *E. coli* DsbC forms a homodimer through a non-thioredoxin dimerization domain linking two Trx-fold domains (the monomeric unit of DsbC is shown for clarity). The secondary structural elements of the Trx fold are highlighted in each case (*red* is used for α-helices and teal for β-sheets, cysteine residues are indicated by *yellow spheres*).

Our detailed knowledge of the properties of isolated cDsbD allowed us to probe the importance of Gln-488 in fine-tuning the electrostatics of this domain and the efficient function of the full-length protein *in vivo*. In wild-type cDsbD_red_, Gln-488, which is distant in the sequence from the -C*XX*C- motif (Fig. 7), is involved in a hydrogen bond network with Asp-455 and Glu-468 and keeps their p*K_a_* values relatively low (p*K_a_* of 5.8 for Asp-455 and even lower for Glu-468). As these acidic residues ionize sequentially, the local concentration of negative charge in the active site pushes the p*K_a_* value of the attacking cysteine (Cys-461) of the -C*XX*C- motif to 10.6 (31).

In this work we have found that replacing Gln-488 with an alanine residue, which cannot form a hydrogen bond or a salt bridge in the active site, has a dramatic effect on the p*K_a_* value of Cys-461. The absence of the stabilizing effect of Gln-488 leads to elevation of the p*K_a_* value of Asp-455 and coupling of the ionization of this acidic residue with the deprotonation of the Cys-461 thiol group. The phenomenon of microscopic p*K_a_* values occurs between Asp-455 and Cys-461 in Q488A-cDsbD_red_ and, as a result, in a fraction of the protein molecule population Cys-461 has a p*K_a_* value of 7.6, which makes it significantly more reactive and prone to reoxidation compared with the wild-type domain. This was apparent when we measured the activity of this variant in the full-length protein *in vivo*; Q488A-DsbD was only ∼75% functional compared with wild-type-DsbD, consistent with part of the protein population being compromised.

The effect of the absence of the Gln-488 side chain in cDsbD could be reversed using a lysine substitution. Gln-488 is not fully conserved in DsbD sequences, and in some organisms an arginine or a lysine can be found in its place. Our choice of the Q488K-cDsbD variant was based on *E. coli* cytoplasmic thioredoxin which has a lysine (Lys-57) with an important role (62) in a structurally equivalent position to Gln-488 in cDsbD. The p*K_a_* value of Cys-461 in Q488K-cDsbD reverts to the wild-type value, and the activity of Q488K-DsbD *in vivo* is comparable (if not slightly enhanced) to the wild-type protein.

##### E. coli Thioredoxin and Human Thioredoxin Also Have Finely Tuned Active Sites Consistent with Their Function

The ionization equilibria of the active-site residues of *E. coli* and human thioredoxin have been the subject of NMR studies since the early 1990s (63, 64). However, measuring the p*K_a_* value of the N-terminal cysteine residue of their -C*XX*C- motifs has proven much more complex than was initially anticipated. The plethora of detailed studies on the thioredoxin systems has been very useful for our work on cDsbD and has made possible the comparative sequence analysis on the Trx fold that follows below.

The active-site residues of cytoplasmic thioredoxin of *E. coli* present a very similar ionization pattern to the one we observed in Q488A-cDsbD. The ionization of Asp-26 is coupled to the deprotonation of Cys-32; microscopic p*K_a_* values (7.5 and 9.2) were observed (15, 61). Asp-26, like Asp-455, is a buried residue and in principle should not lose its proton very readily. However, Lys-57 (structurally equivalent to Gln-488 of cDsbD, see Fig. 7) forms a salt bridge with Asp-26 and stabilizes its carboxylic anion. Absence of Lys-57 leads to elevation of the p*K_a_* value of Asp-26 to 9.4 (62). The stabilization of the Asp-26 anion lowers its p*K_a_* value enough to couple its ionization with the deprotonation of Cys-32. If Asp-26 is absent or if it is not stabilized (in variants lacking Lys-57), the p*K_a_* value of Cys-32 is even higher (∼8) (15, 62, 63). The presence of microscopic p*K_a_* values in *E. coli* thioredoxin is consistent with its role as a bidirectional enzyme. Although its role is primarily that of a reductase, thioredoxin has the capability to also act as an oxidase in more oxidizing environments (65, 66). Part of the thioredoxin molecule population has a high p*K_a_* value, and this protects the reductant load from reoxidation (thus, it can specifically reduce the right substrates), and the remaining population is more prone to reoxidation having a cysteine with a near physiological p*K_a_* value (and this fraction of the protein population can act as a reductant acceptor). Therefore, depending on how oxidizing or reducing the cellular compartment is, there is always a fraction of the thioredoxin molecule population “equipped” to act as an oxidase or reductase, respectively.

The active-site organization of human thioredoxin is different from its *E. coli* equivalent. Asp-26 is present, but there is no positively charged amino acid in the position analogous to Lys-57 of *E. coli* thioredoxin (Fig. 7). Instead, there is a cluster of three acidic residues (Glu-56, Asp-58, and Asp-60 in human thioredoxin-1), which all point toward the active site near this position (PDB entry 1TRS) (52). The p*K_a_* value of Asp-26 in human thioredoxin was unambiguously determined (67) to be 8.1 for the oxidized protein and 9.9 for the reduced state. These elevated p*K_a_* values arise from low accessibility of the carboxyl side chain of Asp-26, hydrogen bonding to the hydroxyl group of Ser-28 (Fig. 7), and the high concentration of negative charge originating from Glu-56, Asp-58, and Asp-60. The p*K_a_* value of Cys-32 in human thioredoxin was measured to be 6.3 (64). The relatively low p*K_a_* value of Cys-32 of human thioredoxin compared with the p*K_a_* value expected for an unperturbed cysteine in a polypeptide (8.5–9.1 (68–70)) is probably due to the presence of the stabilizing helix dipole and possibly to hydrogen bonding of the thiolate to the nitrogen of Cys-35. Thus, the reductive role of this protein is ensured by the lack of stabilizing interactions for the deprotonated form of the aspartic acid six positions before the N-terminal cysteine. The p*K_a_* of Asp-26 is higher in reduced human thioredoxin due to the electrostatic repulsion arising from the nearby thiolate group of Cys-32. In many other Trx folds, it is the electrostatic repulsion arising from the stabilized aspartate side chain that raises the p*K_a_* value of the “attacking” cysteine residue (15, 31).

##### Trx Fold Proteins Have an Extended Active-site Motif

Our own detailed studies on isolated cDsbD and the extensive residue-specific information on *E. coli* and human thioredoxin suggest that the electrostatic balance in the active site of Trx-fold proteins is closely linked to their function. It seems however that this fine-tuning involves a larger part of the protein sequence than just the -C*XX*C- motif. We have performed a comparative sequence analysis of Trx-fold proteins from different organisms (Gram-negative bacteria, Gram-positive bacteria, and eukaryotes), which is shown in Fig. 7. The choice of proteins was dictated by the availability of information about their function, structure, and active-site properties. With the exception of the cysteine pair, which was an obvious choice, the identification of residues that might be of functional importance was based on the primary sequence alignment but also on structural overlays between Trx-fold proteins. This allowed us to identify key amino acids in the vicinity of the -C*XX*C- motif. We checked the sequence conservation of these key residues. The highlighted acidic residues are fully conserved at these positions (either an aspartic or a glutamic acid is found). Residues shown in *cyan* (Fig. 7) are also highly conserved. More specifically, the amino acid found in that position can always perform the same role (stabilizing an anion through a salt bridge or hydrogen bond).

A careful examination of Fig. 7 allows a better understanding of why the function of Trx-fold proteins can be so different and how this diversity is achieved. *E. coli* and human thioredoxin and *E. coli* cDsbD have very different active-site p*K_a_* values consistent with different functions. In all three cases, the aspartic acid six positions prior to the N-terminal cysteine of the -C*XX*C- motif plays a major role. It is worth noting that an acidic residue with very low side chain solvent accessibility is often found in this position in Trx-fold proteins (Fig. 7). In this way, it can act as a switch. In cases where the carboxylic anion of its side chain is not stabilized by an interaction with another residue, the p*K_a_* value of the acid is high and does not affect the p*K_a_* value of the cysteine (for example in human thioredoxin). However, in cases where this anion is stabilized by a hydrogen bond with another side chain (such as Gln-488 in cDsbD) or a salt-bridge interaction (such as Lys-57 in *E. coli* thioredoxin), the p*K_a_* value of the acidic residue is lowered, and the presence of the negative charge near the N-terminal cysteine leads to a higher p*K_a_* value for the latter. It is also the case that in proteins where the N-terminal cysteine of the -C*XX*C- motif does not have a very high p*K_a_* value, it is common to encounter prolines or glycines between the cysteine pair (Fig. 7); these residues may adopt backbone conformations that favor the stabilization of the thiolate of the N-terminal cysteine by a hydrogen bond interaction with the backbone amide of the C-terminal cysteine of the -C*XX*C- motif and possibly other neighboring residues (18).

The N-terminal cysteines of the -C*XX*C- motif of *E. coli* DsbA, *B. subtilis* BdbD, and human protein-disulfide isomerase have been found to have low p*K_a_* values. DsbA is an oxidase, promoting disulfide bond formation in the periplasm of Gram-negative bacteria, with a p*K_a_* value of 3.5 for Cys-30 (71). BdbD is also an oxidase, but it is found in Gram-positive bacteria; the p*K_a_* value of Cys-69 of *B. subtilis* BdbD was determined to be less than 4.5 (50). Protein-disulfide isomerase is a multidomain member of the Trx superfamily consisting of four thioredoxin-like domains. It is responsible for disulfide bond formation, reduction, and isomerization in the endoplasmic reticulum of eukaryotes. The p*K_a_* value of the N-terminal cysteine of the -C*XX*C- motif, which is responsible for disulfide bond formation in the “active” domains, was found to be 4.8 (72). All three proteins have a conserved and completely buried glutamic acid six positions before the N-terminal cysteine of the -C*XX*C- motif (Fig. 7). Like Asp-26 in human thioredoxin, this residue would be expected to have an elevated p*K_a_* value, and thus it would not perturb the p*K_a_* value of the N-terminal cysteine. This elevated p*K_a_* is likely to be due to the low solvent accessibility of its side chain or to electrostatic interactions with residues in α1 and β2 (Fig. 7). In these proteins, an important contribution to the lowering of the cysteine p*K_a_* can be attributed to the helix dipole and the stabilizing hydrogen bonds from the backbone of the -C*XX*C- motif residues (49, 71).

*E. coli* CcmG and *B. subtilis* ResA and StoA are all bacterial reductant provision proteins with a Trx fold. CcmG and ResA provide reductant to apocytochromes before heme attachment (73, 74), whereas StoA is involved in reductant provision during endospore biogenesis (75). All of them have been found to have near physiological p*K_a_* values (∼6–8) for the N-terminal cysteine of their -C*XX*C- motif (50, 76, 77). None of these proteins have an acidic residue at the characteristic position within β1 (Fig. 7, six positions before the N-terminal cysteine of the -C*XX*C- motif). The glutamic acid six positions after the N-terminal cysteine (Fig. 7) in these proteins is highly conserved, but in contrast to the solvent-exposed Glu-468 of cDsbD and Glu-37 of DsbA, it is completely buried. Therefore, it could have a high p*K_a_* value that would not affect the cysteine p*K_a_*. In CcmG, ResA, and StoA, the influence of the helix dipole is probably sufficient to lower the p*K_a_* value of their N-terminal cysteine compared with the p*K_a_* value of a cysteine in a random polypeptide. Near physiological p*K_a_* values are important for reductant provision proteins as their attacking cysteine is nucleophilic enough to carry out reduction of a substrate but not too prone to reoxidation that would lead to a futile cycle.

DsbC is a thiol-disulfide isomerase responsible for the correction of wrongly formed disulfide bonds in the periplasm of Gram-negative bacteria (78, 79). DsbG acts in the same cellular compartment and protects single cysteine residues in proteins from oxidation (80). Both proteins are V-shaped homodimers containing one Trx fold per monomeric unit (56, 57). The formation of the dimer is essential for the function of both proteins, and the presence of a cleft between the monomeric units means that the active site of the Trx-fold domains is contained in a very hydrophobic environment. The *E. coli* DsbC and DsbG sequences are included in Fig. 7 to demonstrate that in this specialized case there are no residues in the structurally equivalent positions of the highlighted amino acids in Fig. 7 that could affect the electrostatic environment of the cysteine pair. No p*K_a_* values have been reported for the cysteines of *E. coli* DsbC or DsbG.

*B. subtilis* BdbA is protein of unknown function, containing a -C*XX*C- motif, and is proposed to have a Trx fold (81). It is included in our comparative sequence analysis to highlight the potential residues that could affect the p*K_a_* value of the N-terminal cysteine. Like CcmG, ResA, and StoA, it lacks the acidic residue on strand β1. However, it does contain a large number of acidic and basic groups located in α1 and β2 (*underlined* in Fig. 7); these could influence the cysteine p*K_a_* values. One could speculate that like CcmG, ResA, and StoA, it could function as a reductase.

##### Concluding Remarks

The Trx fold is abundant in organisms from all kingdoms of life. It has been studied for several decades so there is detailed information on the properties and function of numerous Trx-fold proteins (83). However, it is still not fully understood how the same fold can carry out efficiently several different functions. In this work, we have used high resolution techniques to understand the ionization equilibria in the active site of the C-terminal domain of *E. coli* DsbD. We combined this knowledge with *in vivo* activity assays of the full-length protein and were able to relate the effect of p*K_a_* values on active-site residues to the function of DsbD. Our results, along with comparative sequence analysis of other Trx-fold proteins allowed us to shed light onto the factors that contribute to the functional diversity of Trx-fold proteins.

The comparative analysis presented here shows clearly that the reactivity of Trx-fold proteins is not determined solely by their -C*XX*C- motif. The cysteine p*K_a_* values, which determine reactivity, are fine-tuned by interactions involving several residues distant in sequence but spatially close to the -C*XX*C- motif. We propose that Trx-fold proteins have an extended active-site motif and that residues found in helix α1 and strands β1 and β2 could have a major impact on the p*K_a_* value of the reactive cysteine. The presence of charged groups, the possibility of formation of hydrogen bonds between amino acids, and the low solvent accessibility of residues in this extensive motif optimize the function of the protein. The fact that the reactivity of Trx-fold proteins depends on more residues than just the -C*XX*C- motif adds another level of complexity to the prediction of the function and properties of new proteins belonging to this superfamily.

## Supplementary Material

Supplemental Data

## Footnotes

4 The abbreviations used are:

Trx: thioredoxin
cDsbD: C-terminal domain of DsbD
tmDsbD: transmembrane domain of DsbD
nDsbD: N-terminal domain of DsbD
DTNB: 5,5′-dithiobis-(2-nitrobenzoic acid)
AMS: 4-acetamido-4′-maleimidyl-stilbene-2,2′-disulfonic acid
HSQC: heteronuclear single quantum coherence
TCEP: tris(2-carboxyethyl)phosphine
BisTris: 2-[bis(2-hydroxyethyl)amino]-2-(hydroxymethyl)propane-1,3-diol
PDB: Protein Data Bank.
