# Supplementary material for: An Extended Active-site Motif Controls the Reactivity of the Thioredoxin Fold
Source: J Biol Chem. 2014 Jan 27;289(12):8681–96. doi: 10.1074/jbc.M113.513457 (PMC3961690; doi:10.1074/jbc.M113.513457)
Supplement: Supplemental Data [file supp_289_12_8681__index.html]

An Extended Active-site Motif Controls the Reactivity of the Thioredoxin Fold — An Extended Active-site Motif Controls the Reactivity of the Thioredoxin Fold — Control of Reactivity in the Trx Fold — Supplemental Data 

# An Extended Active-site Motif Controls the Reactivity of the Thioredoxin Fold

## Supplemental Data

**Files in this Data Supplement:**

- Supplemental Tables S1-S5 (.pdf, 179 KB) - Supplemental Tables S1 to S5
