# Supplementary material for: An Extended Active-site Motif Controls the Reactivity of the Thioredoxin Fold
Source: J Biol Chem. 2014 Jan 27;289(12):8681–96. doi: 10.1074/jbc.M113.513457 (PMC3961690; doi:10.1074/jbc.M113.513457)
Supplement: Supplemental Data [file supp_M113.513457_jbc.M113.513457-1.pdf]

SUPPLEMENTAL DATA

An Extended Active-site Motif Controls the Reactivity of the Thioredoxin Fold*

Despoina A. I. Mavridou[1], Emmanuel Saridakis[2], Paraskevi Kritsiligkou[1], Erin C. Mozley[1], Stuart J. Ferguson[1] and Christina Redfield[1]

[1]From the Department of Biochemistry, University of Oxford, South Parks Road, Oxford, OX1 3QU, United Kingdom and the [2]Department of Physical Chemistry, N.C.S.R. Demokritos, Aghia Paraskevi, Athens 15310, Greece

*Running title: *Control of reactivity in the Trx fold*

To whom correspondence should be addressed: Christina Redfield or Despoina A. I. Mavridou, Department of Biochemistry, University of Oxford, South Parks Road, Oxford, OX1 3QU, United Kingdom, Tel: +44(0)1865-275330 (CR), +44(0)1865-613313 (DAIM), E-mail: christina.redfield@bioch.ox.ac.uk or despoina.mavridou@bioch.ox.ac.uk.

SUPPLEMENTAL TABLES

Supplemental Table S1 Plasmids used in this work.

Name	Description	Source
pDzc1	C-terminal domain (T425-P546) of *E. coli* DsbD (cDsbD) with a C-terminal polyhistidine tag, Amp^R	(1)
pDzc1a	pDzc1 with a thrombin-cleavable C-terminal polyhistidine tag, Amp^R	(2)
pDzc10	pDzc1a carrying the Q488A mutation, Amp^R	This work
pDzc12	pDzc1a carrying the Q488K mutation, Amp^R	This work
pDzc13	pDzc1a carrying the D455N/Q488A mutations, Amp^R	This work
pDzn3	N-terminal domain (L2-V132) of *E. coli* DsbD (nDsbD) with a thrombin-cleavable C-terminal polyhistidine tag, Amp^R	(3)
pDsbd1	Full-length *E. coli* DsbD C-terminal streptavidin II tag, Amp^R	(3)
pDsbd3	pDsbd1 carrying the C464A mutation, Amp^R	(3)
pDsbd7	pDsbd1 carrying the Q488A mutation, Amp^R	This work
pDsbd10	pDsbd1 carrying the Q488K mutation, Amp^R	This work
pRZ001	*P. denitrificans* cytochrome cd_1, $Gent^R$	Lab stock
pEC86	*E. coli ccmABCDEFGH*, Cam^R	(4)

Supplemental Table S2 Oligonucleotide primers for site-directed mutagenesis used in this work. The mutated codon is in bold.

Name	DNA sequence (5´-3´)	Resulting plasmids
DM1	GACACGGTCTTACTT**GCG**GCCAACGTCACGGCC	pDzc10, pDsbd7
DM2	GGCCGTGACGTTGGC**CGC**AAGTAAGACCGTGTC	pDzc10, pDsbd7
DM3	GACACGGTCTTACTT**AAG**GCCAACGTCACGGCC	pDzc12, pDsbd10
DM4	GGCCGTGACGTTGGC**CTT**AAGTAAGACCGTGTC	pDzc12, pDsbd10
DM5	CCGGTGATGTTA**AAT**CTTTATGCCGACTG	pDzc13
DM6	CAGTCGGCATAAAG**ATT**TAACATCACCGG	pDzc13

Supplemental Table S3 Bacterial strains used in this work.

Name	Description	Source
MC1000	*ara*D139, Δ(*ara*, *leu*)7697, Δ*lac*X74, *gal*U, *gal*K, *str*A	(5)
MC1000 (Δ*dsbD*)	MC1000 Δ*dsbD*	(6)
BL21 (DE3)	F–, *omp*T, *hsd*S_B(r_B-, m_B-), *dcm*, *gal*, λ(DE3)	Stratagene

Supplemental Table S4 Results from the fitting of the pH dependence of the chemical shifts of the $^{13}C^{\beta}$ and $^{1}H^{\beta}$ of C461 and C464 in wild-type and mutant oxidized and reduced cDsbD.

WT-cDsbD

	Oxidized						Reduced					
	C461			C464			C461			C464		
	$^{13}C^{\beta}$	$H^{\beta1}$	$H^{\beta2}$	$^{13}C^{\beta}$	$H^{\beta1}$	$H^{\beta2}$	$^{13}C^{\beta}$	$H^{\beta1}$	$H^{\beta2}$	$^{13}C^{\beta}$	$H^{\beta1}$	$H^{\beta2}$
pK_1	6.8	-	-	-	6.7	6.8	10.5	6.0	10.6	5.9	5.6	5.7
pK_2	-	-	-	-	-	-	-	10.4	-	10.9	10.5	10.6
δ_{HAH}	43.0	-	-	-	3.94	3.23	26.7	2.39	2.32	26.8	3.23	3.05
δ_{HA^-}	42.6	-	-	-	3.74	3.46	29.3	2.56	2.27	27.1	3.32	3.18
$\delta_{A^{2-}}$	-	-	-	-	-	-	-	2.61	-	27.7	2.85	3.18

Q488A-cDsbD

	Oxidized						Reduced					
	C461			C464			C461			C464		
	$^{13}C^{\beta}$	$H^{\beta1}$	$H^{\beta2}$	$^{13}C^{\beta}$	$H^{\beta1}$	$H^{\beta2}$	$^{13}C^{\beta}$	$H^{\beta1}$	$H^{\beta2}$	$^{13}C^{\beta}$	$H^{\beta1}$	$H^{\beta2}$
pK_1	6.2	-	8.5	8.2	8.1	7.9	7.7	7.7	6.6	7.6	7.9	6.3
pK_2	8.2	-	-	-	-	-	10.5	10.0	-	10.7	10.5	10.5
δ_{HAH}	43.7	-	3.18	33.9	4.11	3.33	26.7	2.58	2.39	27.3	3.30	3.25
δ_{HA^-}	43.4	-	3.10	33.5	3.84	3.49	27.6	2.63	2.30	27.7	3.21	3.14
$\delta_{A^{2-}}$	42.7	-	-	-	-	-	29.5	2.69	-	28.5	2.90	3.40

D455N/Q488A-cDsbD

	Oxidized						Reduced					
	C461			C464			C461			C464		
	$^{13}C^{\beta}$	$H^{\beta1}$	$H^{\beta2}$	$^{13}C^{\beta}$	$H^{\beta1}$	$H^{\beta2}$	$^{13}C^{\beta}$	$H^{\beta1}$	$H^{\beta2}$	$^{13}C^{\beta}$	$H^{\beta1}$	$H^{\beta2}$
pK_1	-	-	-	-	-	-	8.8	8.8	8.6	8.9	8.8	9.0
pK_2	-	-	-	-	-	-	-	-	-	-	-	-
δ_{HAH}	-	-	-	-	-	-	26.6	2.30	2.50	27.1	3.32	3.03
δ_{HA^-}	-	-	-	-	-	-	29.5	2.22	2.60	28.9	2.81	3.59
$\delta_{A^{2-}}$	-	-	-	-	-	-	-	-	-	-	-	-

Q488K-cDsbD

	Oxidized						Reduced					
	C461			C464			C461			C464		
	$^{13}C^{\beta}$	$H^{\beta1}$	$H^{\beta2}$	$^{13}C^{\beta}$	$H^{\beta1}$	$H^{\beta2}$	$^{13}C^{\beta}$	$H^{\beta1}$	$H^{\beta2}$	$^{13}C^{\beta}$	$H^{\beta1}$	$H^{\beta2}$
pK_1	8.2	-	8.5	8.2	8.0	8.1	8.1	-	7.9	7.6	10.4	7.0
pK_2	-	-	-	-	-	-	10.3	-	-	10.5	-	10.3
δ_{HAH}	43.0	-	2.98	33.5	3.88	3.25	26.7	-	2.40	26.9	3.24	3.04
δ_{HA^-}	42.7	-	2.90	33.3	3.75	3.51	27.0	-	2.56	27.3	2.74	2.89
$\delta_{A^{2-}}$	-	-	-	-	-	-	29.3	-	-	28.0	-	3.26

Supplemental Table S5 Results from the fitting of the pH dependence of the chemical shifts of the ^{15}N and $^{1}H^{N}$ of D455 in wild-type and mutant oxidized and reduced cDsbD.

**D455 in WT-cDsbD**				
	**Oxidized**		**Reduced**	
	^{15}**N**	^{1}**H**^{N}	^{15}**N**	^{1}**H**^{N}
**p*K*$_1$**	6.6	-	5.9	-
**p*K*$_2$**	-	-	10.5	-
δ_{HAH}	125.8	-	126.4	-
δ_{HA^-}	127.4	-	127.6	-
δ_{A2^-}	-	-	128.0	-
**D455 in Q488A-cDsbD**				
	**Oxidized**		**Reduced**	
	^{15}**N**	^{1}**H**^{N}	^{15}**N**	^{1}**H**^{N}
**p*K*$_1$**	7.7	7.5	7.3	6.8
**p*K*$_2$**	-	9.8	10.4	8.9
δ_{HAH}	126.3	9.18	126.6	9.17
δ_{HA^-}	127.5	8.89	127.3	8.95
δ_{A2^-}	-	8.82	128.6	8.88
**D455 in Q488K-cDsbD**				
	**Oxidized**		**Reduced**	
	^{15}**N**	^{1}**H**^{N}	^{15}**N**	^{1}**H**^{N}
**p*K*$_1$**	8.0	8.0	7.2	7.1
**p*K*$_2$**	-	-	10.9	-
δ_{HAH}	125.7	9.14	125.7	9.13
δ_{HA^-}	126.8	8.84	126.9	8.84
δ_{A2^-}	-	-	127.7	-

REFERENCES

1. Bushell, K. M., Ferguson, S. J., and Redfield, C. (2002) ^{1}H, ^{15}N and ^{13}C assignments of the carboxy-terminal domain of the transmembrane electron transfer protein DsbD. *J. Biomol. NMR* **24**, 359-360
2. Mavridou, D. A., Stevens, J. M., Ferguson, S. J., and Redfield, C. (2007) Active-site properties of the oxidized and reduced C-terminal domain of DsbD obtained by NMR spectroscopy. *J. Mol. Biol.* **370**, 643-658
3. Mavridou, D. A., Saridakis, E., Kritsiligkou, P., Goddard, A. D., Stevens, J. M., Ferguson, S. J., and Redfield, C. (2011) Oxidation state-dependent protein-protein interactions in disulfide cascades. *J. Biol. Chem.* **286**, 24943-24956
4. Arslan, E., Schulz, H., Zufferey, R., Kunzler, P., and Thony-Meyer, L. (1998) Overproduction of the *Bradyrhizobium japonicum* *c*-type cytochrome subunits of the cbb_3 oxidase in *Escherichia coli*. *Biochem. Biophys. Res. Commun.* **251**, 744-747
5. Casadaban, M. J., and Cohen, S. N. (1980) Analysis of gene control signals by DNA fusion and cloning in *Escherichia coli*. *J. Mol. Biol.* **138**, 179-207
6. Stewart, E. J., Katzen, F., and Beckwith, J. (1999) Six conserved cysteines of the membrane protein DsbD are required for the transfer of electrons from the cytoplasm to the periplasm of *Escherichia coli*. *EMBO J.* **18**, 5963-5971
